# Quantitative Analysis of Berberidis Cortex via Quantitative Analysis of Multicomponents by Single Marker (QAMS) Combined with Fingerprint and Chemometrics Methods

**DOI:** 10.1155/2022/8042631

**Published:** 2022-08-25

**Authors:** Yuting Su, Yao Peng, Jie Ren, Shangjie Wu, Si Lei, Fei Peng, Zhina Sun, Xiuqing He, Juan Li, Shunxiang Li

**Affiliations:** ^1^Hunan Engineering Technology Research Center for Bioactive Substance Discovery of Chinese Medicine, School of Pharmacy, Hunan University of Chinese Medicine, Changsha 410208, China; ^2^Hunan Province Sino-US International Joint Research Center for Therapeutic Drugs of Senile Degenerative Diseases, Changsha 410208, China; ^3^The Second XiangYa Hospital, Central South University, Changsha 410008, China; ^4^Qinghai Red Cross Hospital, Xining 810099, China

## Abstract

Berberidis Cortex is rich in alkaloids, and many of them have antibacterial, anti-inflammatory, and hypoglycemic activities. However, few research studies have focused on the quantitative analysis of multiple components from Berberidis Cortex. In this study, a new quality evaluation strategy for Berberidis Cortex was developed and validated by high-performance liquid chromatography (HPLC), which involved single marker, fingerprint, and stoichiometric methods. Using berberine hydrochloride as an internal reference, the relative correction factors of palmatine hydrochloride, magnoline, and jatrorrhizine hydrochloride were 2.4537, 0.9783, and 1.0035, respectively, and their durabilities were also well performed. In addition, both methods mentioned above were used to compare the mass fractions of four isoquinoline alkaloids in ten batches of Berberidis Cortex from different origins. These results indicated that the approach applied in this study was accurate and feasible. The fingerprints of these ten batches of Berberidis Cortex were established, and eleven components were identified with the similarity greater than 0.993. Both cluster and principal component analysis were carried out based on the peak area of these components, the results demonstrated that these ten batches of Berberidis Cortex were divided into two groups and the distribution of the medicinal material was basically consistent. Therefore, quantitative analysis of multicomponents by single marker (QAMS) can be widely used in the quality control of Berberidis Cortex as theoretical basis.

## 1. Introduction

**Figure 1:**
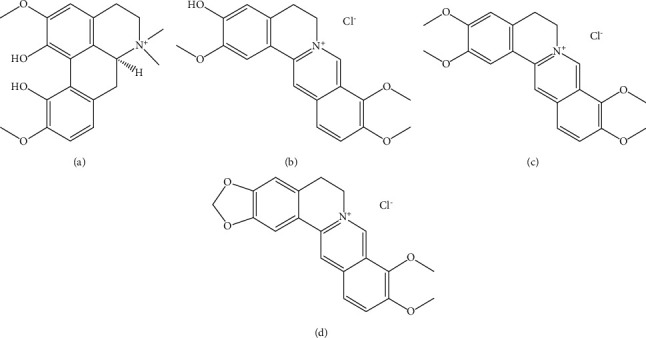
The chemical structure of four alkaloid compounds. (a) Magnoflorine; (b) jatrorrhizine hydrochloride; (c) palmatine hydrochloride; (d) berberine hydrochloride.

Berberidis Cortex is the dry bark of *Berberidis kansuensis* Schneid, or other plants of the same species. As a typical traditional Chinese medicine (TCM), Berberidis Cortex is widely used in Tibetan medicine. Berberidis Cortex mainly distributed in Qinghai, Gansu, Sichuan, and Shanxi provinces of China, and Berberidis Cortex was first recorded in the famous Tibetan medicine book “The Four Medical Tantras,” stating that it has significant effects on the treatment of dysentery, urinary tract infection, nephritis, and conjunctivitis. In recent studies, researches have proved that the alkaloids are the type of natural products with various bioactivities [1–6], such as antibacterial, anti-inflammatory, antiviral, antimalarial, antidiabetic, and others. Berberidis Cortex exhibited similar bioactivities [4, 7–14]. Various alkaloids were found from it, such as berberine hydrochloride, palmatine hydrochloride, magnoline, and jatrorrhizine hydrochloride (Figure 1) [15–19].

TCM consists of diverse components and even their mixtures [20]. To date, Berberidis Cortex has been recorded in many local standards in China. However, most of them only rely on one single active substance (berberine hydrochloride) has been used by thin layer chromatography for its quality control. Thus, developing efficient and comprehensive methods to determine various bioactive substances and evaluate the quality standard of Berberidis Cortex is necessary. Quantitative analysis of multicomponents by single marker (QAMS) is a novel method that has been widely applied for the quality evaluation of TCM, which requires only one single reference standard to determine multiple components simultaneously by the intrinsic functional relationship among the bioactive compounds in TCM [21–25]. Compared with the traditional external standard method (ESM), QAMS can reduce the experimental cost and shorten the detection cycle [26]. Due to these advantages, six Chinese herbs are included in the Chinese Pharmacopoeia (2020 edition, volume I) by using simultaneous determination of multicomponents via QAMS approach, such as *Coptides rhizoma*, *Salvia miltiorrhiza, Bofonis corium,* and *Ginger*. Moreover, many other countries have also accepted this proposal [27, 28]. As an effective method for quality evaluation of herbs and their relevant products, fingerprint analysis has been accepted by the WHO, the FDA, and the State Food and Drug Administration of China which focuses on the characterization of the overall sample composition [29–31]. Similarity assessment between batches of different origin can reflect the consistency of origin and chemical composition of samples. Chemometric methods, such as clustering analysis (CA) and principal component analysis (PCA), called unsupervised chemometrics, can be used for comprehensive evaluation of different varieties of Berberidis Cortex, in order to provide reference for primitive identification and quality control [32, 33].

In this work, we aimed at establishing an effective and sensitive method by QAMS to simultaneously determine four active alkaloid substances in the Tibetan medicine Berberidis Cortex, namely, berberine hydrochloride, palmatine hydrochloride, magnoline, and jatrorrhizine hydrochloride. Meanwhile, the chemometrics methods, CA and PCA, uniting with HPLC fingerprint were all applied together in the source and chemical composition analysis, which comprehensively reflected the differences among ten batches of Berberidis Cortex. To our best knowledge, this is the first time that the QAMS method, fingerprint analysis, and chemometrics methods are combined together for the quality evaluation of Berberidis Cortex. These results also provide a theoretical basis for achieving the quality control standards of Berberidis Cortex and its preparations.

## 2. Materials and Methods

### 2.1. Chemicals and Reagents

All of the standard substances, jatrorrhizine hydrochloride (110733–201609, purity 89.5%), palmatine hydrochloride (110732–201913, purity 85.7%), and berberine hydrochloride (110713–202015, purity 85.9%), were purchased from National Institute for Food and Drug Control. The magnoflorine (P23J11L119379, purity 98.0%) was purchased from Shanghai-Yuanye Biotechnology Co., Ltd. (Shanghai, China). Chromatographical-grade acetonitrile was provided by TEDIA Co., Ltd. (Ohio, United States). All other reagents used were analytical grade and supplied by Sinopharm Chemical Reagent Co., Ltd. (Shanghai, China). Ultrapure water was used during the experiment.

### 2.2. Plant Materials

Ten batches of Berberidis Cortex were manufactured by Qinghai-Xueyu Chinese-Tibetan Herbal Pieces Processing Co., Ltd. (Qinghai, China) at different seasons from Qinghai provinces in China (Table 1).

### 2.3. Instrument and Chromatographic Conditions

The chromatographic system was performed on Waters e2695 HPLC (Waters, United States) and Waters ACQUITY Arc e2695 UHPLC (Waters, United States), both equipped with Empower chromatographic working station. The following other instruments were used: SB-5200DTD ultrasonic machine (Scientz, Ningbo, China); BSA124S-CW electronic analytical balance (Sartorius, Germany); JS15-01 electronic analytical balance (Shanghai-Puchun, China).

The Phenomenex-Luna C_18_ (250 × 4.6 mm, 5 *μ*m), CAPCELL PAK C_18_ (250 × 4.6 mm, 5 *μ*m), and Venusil XPB C_18_ (250 × 4.6 mm, 5 *μ*m) chromatographic columns were adopted during the analysis. The mobile phase consisted of acetonitrile (solvent A) and 0.3% phosphoric acid solution mixed with triethylamine to pH 2.5 (solvent B). The gradient elution was as followed: 10–17% A for 0–10 min; 17–28% A for 10–20 min; 28–30% A for 20–30 min; 30–34% A for 30–35 min. The flow rate was set at 1.0 mL/min, and the sample injection volume was 10 *μ*L; The column temperature was 30°C, and the detection wavelength was set at 270 nm.

### 2.4. Preparation of Standard Solutions

Four standard substances were weighed accurately and dissolved by 10 mL of methanol in the volumetric flask. The concentration of mixed-standard solutions was as followed: 1.5 mg/mL of magnoflorine, 0.87 mg/mL of jatrorrhizine hydrochloride, 0.76 mg/mL of palmatine hydrochloride, and 1 mg/mL of berberine hydrochloride. Then, the stock solution was diluted into five different concentrations for the linearity experiment. All the standard solutions were stored in the refrigerator at 0–4°C for later use.

### 2.5. Preparation of Sample Solutions

Ten batches of Berberidis Cortex were pulverized and screened through a 60-mesh sieve, respectively. The sample solutions were prepared by ultrasonic extraction. Firstly, 0.5 g of each sample powder was accurately weighed and added into a glass-stopper Erlenmeyer flask with 20.0 mL of 80% methanol. Secondly, the mixture was ultrasonicated at 30°C for 20 minutes. After the mixture was cooled to room temperature, 80% methanol was used to fill the lost weight. Finally, the sample solutions were filtered through 0.22 *μ*m filters before HPLC analysis.

### 2.6. Data Analysis

The fingerprint similarity of Berberidis Cortex was evaluated by Similarity Evaluation System for the chromatographic fingerprint of TCMs (version 2012, Chinese Pharmacopoeia Committee). The standardized fingerprint chromatograms were obtained based on the calibration and normalization of the common peak in ten batches of Berberidis Cortex SIMCA (14.1 Version, Umetrics, Sweden) multivariate statistical analysis software was used for Principal component analysis (PCA) by common peak area in ten batches. Taking the peak area as variables, the clustering analysis (CA) was calculated by SPSS statistical analysis software (21.0 Version, IBM Corp, United States) and GraphPad Prism (6.01 Version) for sample classification. Besides, verification of the accuracy and reliability of QAMS by comparing with ESM was performed to determine the other four active components in samples.

## 3. Results

### 3.1. Calibration Curves

The calibration curves were established by measuring the mixed-standard solutions with six different concentrations. As Table 2 shown, four calibration curves represented excellent linearity with high squared correlation coefficient values (*R*^2^ ≥ 0.9999) within the detected range. The limit of detection (LOD) and limit of quantification (LOQ) of each alkaloid were defined as signal-to-noise ratio (S/N) of 3 and 10. For the four compounds' determination, the LOD and LOQ ranged from 0.00436 to 0.0113 mg/mL and from 0.01454 to 0.03768 mg/mL, respectively.

### 3.2. Method Validation

**Figure 2:**
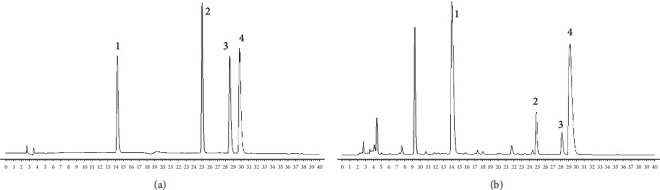
Chromatograms of the standard solutions and sample solutions. (a) Mixed standard solution; (b) sample solution; (1) magnoflorine; (2) jatrorrhizine hydrochloride; (3) palmatine hydrochloride; (4) berberine hydrochloride.

The precision, stability, and repeatability were tested and analyzed to validate the method properties, and the chromatograms are presented in Figure 2. The same mixed-standard solution of 10 *μ*l was injected for six consecutive times under chromatographic conditions, and their RSDs were calculated. The RSD rates ranging from 0.08 to 0.15% indicated that the proposed method is appropriate for analytes quantification. In the stability experiment, the prepared mix-standard solution was, respectively, detected at 0, 4, 6, 8, 10, 12, and 24 hours at room temperature. The RSD values were between 1.5 and 1.9%, which proved that the sample solutions represented high stability within 24 hours. To confirm the repeatability of the method, six independently prepared solutions from the same batch were analyzed. The RSD of magnoflorine, jatrorrhizine hydrochloride, palmatine hydrochloride, and berberine hydrochloride was 1.8%, 1.1%, 1.9%, and 1.8%, and the average mass concentrations of four alkaloids were 65.7280 mg/g, 4.1964 mg/g, 2.7840 mg/g, and 30.6083 mg/g, respectively. The results show that the proposed method represented high repeatability. To verify the accuracy of the method, four standard substances shown above were precisely added in 0.1 g powder of Berberidis Cortex, and the samples were prepared by the method mentioned above. The average recovery rate (*n* = 6) of the peak area were ranging from 102.81 to 104.91%.

### 3.3. Quantitative Analysis of Multicomponents by the Single Marker

#### 3.3.1. Calculate Relative Correction Factors and Relative Retention Time

The relative correction factors (RCF) were determined by multipoint correction and injected the 1, 2, 4, 6, 8, and 10 *μ*L of mixed-standard solutions volumes. Berberine hydrochloride was chosen as the internal referring standard based on its stability, accessibility, and pharmacological activity. The RCF values were shown in Table 3, and calculated by the following formula:

(1) $$\begin{array}{c} f_{\left(s/i\right)}\frac{f_{s}}{f_{i}}=\frac{A_{s}\times C_{i}}{A_{i}\times C_{s}}, \end{array}$$

where *A*_*s*_ is the peak area of internal standard (berberine hydrochloride); *A*_*i*_ is the peak area of the sample component; *C*_*s*_ is the concentration of internal standard solution; *C*_*i*_ is the concentration of the sample solution. The RCFs for the four compounds, magnoflorine, jatrorrhizine hydrochloride, and palmatine hydrochloride, were 1.2426, 0.9905, and 0.5350 with the range of RSD values from 0.38% to 0.99%, respectively.

The relative retention time (RRT) was used for locating the target components in chromatography by using the different chromatographic columns and instruments. The RRT values were calculated by the formula as follows:

(2) $$\begin{array}{c} t_{R=\frac{t_{R\left(i\right)}}{t_{R\left(s\right)}}}\prime \end{array}$$

where *t*_*R*(*i*)_ is the retention time of the sample component; *t*_*R* (*s*)_ is the retention time of internal standard (berberine hydrochloride). As the results are shown in Table 4, the RRT of magnoflorine, jatrorrhizine hydrochloride, and palmatine hydrochloride were 0.4784, 0.8494, and 0.9672, respectively, with the RSD range from 0.41% to 2.8%.

### 3.4. Durability Measurements of the RCFs

To verify the durability of the QAMS method, the chromatographic columns, columns temperature, and chromatographic instruments, the RCFs of mixed-standard solution were tested and analyzed. As presented in Table 5, the RSD rate of different factors ranged from 0.27% to 1.4%, indicating that the proposed method possessed great durability in the detection.

### 3.5. Comparison of the Results of the External Standard Method (ESM) with QAMS

To systematically evaluate the feasibility of QAMS, the mass fractions of four target alkaloids in the samples from ten batches of Berberidis Cortex were compared by ESM and QAMS (Table 6). The comparison of the two methods was based on the standard method difference (SMD). The absolute values of SMD were less than 2%, which indicated that the QAMS method is feasible to simultaneously determine four alkaloidal components in Berberidis Cortex. The SMD values were calculated by the following equation:

(3) $$\begin{array}{c} \text{SMD}\left(\%\right)=\frac{W_{\text{ES }}-W_{\text{QAMS}}}{W_{\text{ES}}}\times 100\%\text{ES }, \end{array}$$

where *W*_ES_ and *W*_QAMS_ is the mass concentrations of compound calculated by ESM and QAMS methods.

### 3.6. Fingerprint Analysis and Similarity Analysis

**Figure 3:**
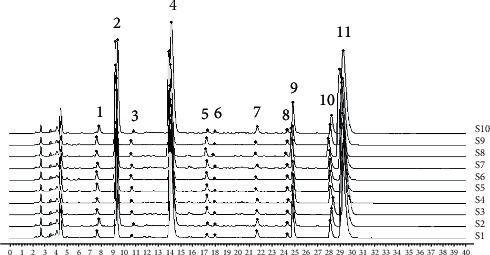
HPLC fingerprints of the ten batches of Berberidis Cortex.

The fingerprint chromatograph of ten batches of Berberidis Cortex were established by the Similarity Evaluation System for the chromatographic fingerprint of TCMs (Version 2012, Chinese Pharmacopoeia Committee) with the multipoint correction and 0.1 of the time window. As presented in Figure 3, the chromatograms were overlaid and aligned, which identified 11 common peaks. The proposed method's similarity was greater than 0.993, which indicated that ten batches of samples shared high similarity. According to the comparison of retention times, the 4^th^, the 9^th^, the 10^th^, and the 11^th^ peaks were identified as magnoflorine, jatrorrhizine hydrochloride, and palmatine hydrochloride.

### 3.7. Clustering Analysis

**Figure 4:**
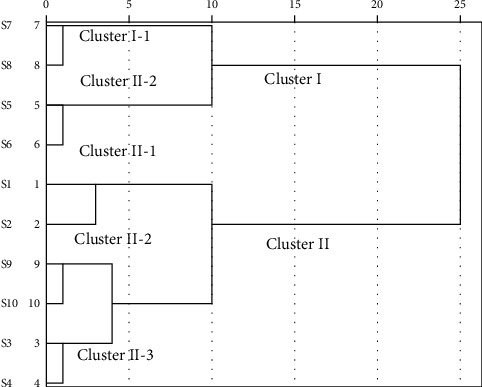
Clustering analysis of ten batches of Berberidis Cortex from different origins.

**Figure 5:**
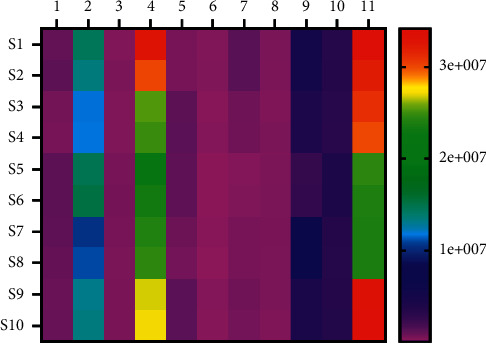
The heat map analysis of ten batches of Berberidis Cortex.

Clustering analysis (CA), a multivariate analysis technique, was widely applied in sample origin classification. By using the 11 characteristic peaks area as the clustering variable, the samples' differences and resemblance characteristics were calculated and analyzed. As Figure 4 shows, when the Euclidean distance was set at 15, the ten batches of Berberidis Cortex were categorized into two clusters. Cluster I contained S5, S6, S7, and S8, which came from Qilian and Tongran in Qinghai province. Cluster II included S1, S2, S3, S4, S9, and S10, which came from Xining, Huangnan, Qilian, and Zeku in Qinghai province. When the Euclidean distance was chosen at 0 to 5, the same origin samples with different seasons were classified into Cluster I-1, Cluster I-2, Cluster II-1, Cluster II-2, and Cluster II-3. The result indicated that similar climate and origin areas might be responsible for the similar chemical components of TCM herbs. Furthermore, the heat map (Figure 5) of Berberidis Cortex also indicated the same classification information. The 4^th^ and the 11^th^ peaks of each batch in Cluster I and Cluster II displayed with similar color, respectively, which is consistent with the CA.

### 3.8. Principal Component Analysis

**Figure 6:**
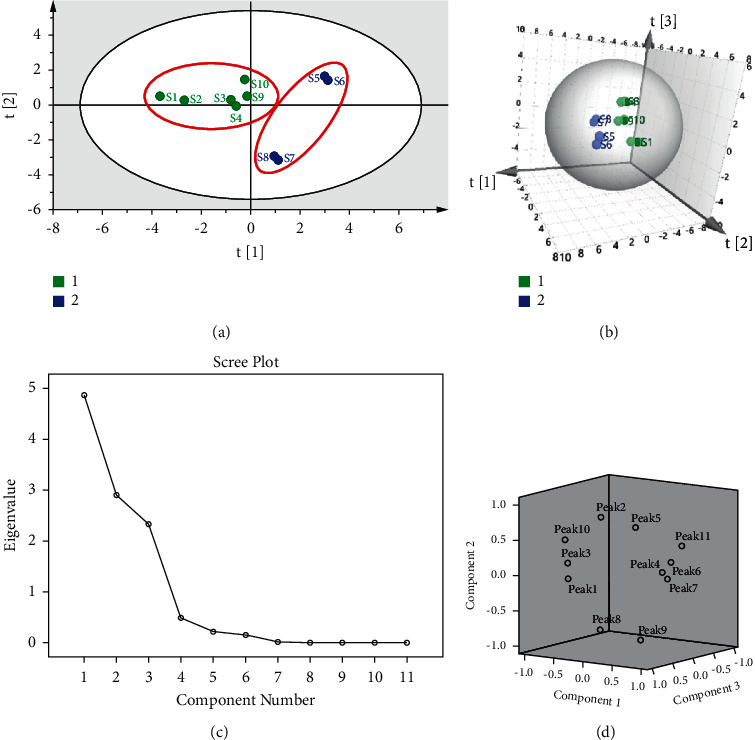
(a) The PCA score scatter plots of samples. (b) The PCA scatter 3D plot of samples. (c) Scree plot. (d) Samples in planar distribution of three dimensions

PCA is an unsupervised analysis method that reduces data dimensionality by extracting principal components and simplifying the data to the greatest extent while reducing information loss. Ten batches of Berberidis Cortex were analyzed and classified by PCA. As shown in Figure 6(a) and 6(b), the results of scatter plots and 3D plots of samples were consistent with CA, which indicated that the results of cluster analysis and PCA could be mutually authenticated. The top three principal components (PC1, PC2, and PC3) contained the most information of all variables, and the cumulative contribution of the top three components was 91.89%. The total variance of PC1, PC2, and PC3 were 44.26%, 26.42%, and 21.21%, respectively (Figure 6(c) and Table 7). Combining Figure 6(d) and the above data show that the superposition effect of multiple components was the cause of the difference in the chemical composition of Berberidis Cortex.

## 4. Discussion

QAMS is widely designed for quality evaluation of TCM and its related products. In this study, taking the berberine hydrochloride as the internal standard, the four components of Berberidis Cortex were precisely quantified using the QAMS method. The method validation results also indicated the feasibility, sensitivity, and accuracy of Berberidis Cortex's quality evaluation. The fingerprint was combined with the similarity analysis and other chemometrics methods to classify the Berberidis Cortex from different regions, which verified that ten batches of Berberidis Cortex were categorized into two clusters and indicated that different origins and seasons might have an impact on the intrinsic quality of TCM herbs. Meanwhile, the results of the chemometrics analysis could be mutually authenticated. Berberidis Cortex is a well-known TCM in Tibetan, in which the quality standard only exists in a few local standards and focuses on a single component. However, TCM exerts the therapeutic effect through multiple components and multiple targets, which shows that one single index to consider the quality of TCM is imprecise. The determination of the multiple active compounds of Berberidis Cortex is not developed yet, which could not evaluate the quality scientifically. In this work, an analytical method was established for the content of four alkaloids in Berberidis Cortex by QAMS.

Furthermore, fingerprint and chemometrics could develop a feasible way to accurately evaluate and classify the quality in different batches of Berberidis Cortex. These will serve as efficient and valuable methods for evaluating Berberidis Cortex's quality or other related products. At the same time, it also provides a specific scientific data foundation for its in-depth study of quality standards.

## Figures and Tables

**Table 1:** The source information of the samples.

Number	Origins	Date of collection
S1	Xining, Qinghai	2019.06
S2	Xining, Qinghai	2019.10
S3	Huangnan, Qinghai	2020.08
S4	Huangnan, Qinghai	2020.10
S5	Qilian, Qinghai	2020.08
S6	Qilian, Qinghai	2020.10
S7	Tongren, Qinghai	2020.09
S8	Tongren, Qinghai	2020.10
S9	Zeku, Qinghai	2020.09
S10	Zeku, Qinghai	2020.10

**Table 2:** Calibration curves of four alkaloids of the reference substance.

Analytes	Regression equation	R	LOD (mg/mL)	LOQ (mg/mL)	Linear range (mg/mL)
Magnoflorine	*y* = 15369*x* + 153559	0.9999	0.0113	0.0377	0.0780∼1.550
Jatrorrhizine hydrochloride	*y* = 38648*x* + 192212	0.9999	0.0056	0.0185	0.0440∼0.8700
Palmatine hydrochloride	*y* = 37947*x* + 146764	0.9999	0.0055	0.0182	0.0380∼0.7600
Berberine hydrochloride	*y* = 39003*x* + 87162	1.000	0.0044	0.0145	0.0500∼1.000

**Table 3:** Taking the berberine hydrochloride as the internal standard to calculate the RCF values.

Injection volume (*μ*L)	*f*_*s*/*A*_	*f*_*s*/B_	*f*_*s*/C_
10	2.4742	0.9914	1.0084
8	2.4476	0.9837	1.0039
6	2.4480	0.9831	0.9975
4	2.4433	0.9768	0.9990
2	2.4476	0.9664	1.0047
1	2.4614	0.9682	1.0072
Mean	2.4537	0.9783	1.0035
RSD (%)	0.48	0.99	0.38

*f*_s/A_: *f*_Berberine hydrochloride/magnoflorine_; *f*_*s*/B_: *f*_Berberine hydrochloride/jatrorrhizine hydrochloride_; *f*_*s*/C_: *f*_Berberine hydrochloride/palmatine hydrochloride_.

**Table 4:** Taking the berberine hydrochloride as the internal standard to calculate the RRT values.

Factors	Chromatographic columns	*f*_s/A_	*f*_*s*/B_	*f*_*s*/C_
Waters 2695	CAPCELL PAK C_18_	0.4970	0.8434	0.9621
	Phenomenex luna C_18_	0.4802	0.8497	0.9655
	Venusil XBP C_18_	0.4910	0.8530	0.9664
Waters acquity	CAPCELL PAK C_18_	0.4694	0.8463	0.9675
	Phenomenex luna C_18_	0.4693	0.8463	0.9675
	Venusil XBP C_18_	0.4638	0.8576	0.9741
Mean		0.4784	0.8494	0.9672
RSD (%)		2.8	0.61	0.41

**Table 5:** The RCF values of three alkaloids in different conditions.

Chromatographic condition	Factors	Chromatographic columns	*f*_s/A_	*f*_*s*/B_	*f*_*s*/C_
Column	Waters 2695	CAPCELL PAK C_18_	2.4867	0.9971	1.0096
		Phenomenex luna C_18_	2.5126	0.9975	1.0179
		Venusil XBP C_18_	2.4742	0.9914	1.0077
	Waters ACQUITY	CAPCELL PAK C_18_	2.4459	0.9849	0.9938
		Phenomenex luna C_18_	2.4908	0.9825	1.0169
		Venusil XBP C_18_	2.4175	0.9762	0.9867
	Mean		2.4713	0.9883	1.0054
	RSD (%)		1.4	0.86	1.3
Column temperature	20°C		2.4394	0.9840	1.0026
	25°C		2.4489	0.9962	1.0028
	30°C		2.4447	0.9876	1.0037
	35°C		2.4315	0.9683	0.9978
	Mean		2.4411	0.9840	1.002
	RSD (%)		0.31	1.2	0.27

**Table 6:** Comparision of the results from the ESM and QAMS (mg g^−1^) (*n* = 3).

Sample	Magnoflorine (mg·g^−1^)		Jatrorrhizine hydrochloride (mg·g^−1^)		Palmatine hydrochloride (mg·g^−1^)		Berberine hydrochloride (mg·g^−1^)
	ESM	QAMS	ESM	QAMS	ESM	QAMS	ESM
S1	72.054	72.997	4.4788	4.4383	3.0232	3.0468	30.684
S2	69.421	70.329	4.7649	4.7155	3.2925	3.3037	30.551
S3	59.079	59.852	3.9308	3.8901	3.0882	3.0987	29.654
S4	57.634	58.390	3.8432	3.8034	3.029	3.0393	28.76
S5	55.062	55.783	2.5538	2.5274	4.0719	4.0858	25.312
S6	55.69	56.418	2.6996	2.6717	4.0044	4.018	23.541
S7	57.297	58.046	6.8179	6.7473	3.4486	3.4603	23.705
S8	57.608	58.362	6.9338	6.862	3.3088	3.3200	23.197
S9	60.988	61.786	4.0477	4.0058	3.2511	3.2621	31.403
S10	62.747	63.569	4.0798	4.0376	3.3541	3.3655	32.401

**Table 7:** Characteristic value and variance contribution rate.

Component	Initial eigenvalues			Extraction sums of squared loadings		
	Total	% of variance	Cumulative %	Total	% of variance	Cumulative %
1	4.869	44.260	44.260	4.869	44.260	44.260
2	2.906	26.420	70.679	2.906	26.420	70.679
3	2.333	21.210	91.890	2.333	21.210	91.890

## Data Availability

All the data, models, and code generated or used during the study appear in the submitted article.

## Abbreviations

TCM: Traditional Chinese medicine
QAMS: Quantitative analysis of multicomponents by single marker
ESM: External standard method
CA: Clustering analysis
PCA: Principal component analysis
LOD: Limit of detection
LOQ: Limit of quantification
S/N: Signal-to-noise ratio
RSD: Relative standard deviation
RCF: Relative correction factors
RRT: Relative retention time
SMD: Standardized mean difference.
